# Shaping tomorrow’s support: baseline clinical characteristics predict later social functioning and quality of life in schizophrenia spectrum disorder

**DOI:** 10.1007/s00127-024-02630-4

**Published:** 2024-03-08

**Authors:** Jiasi Hao, Natalia Tiles-Sar, Tesfa Dejenie Habtewold, Edith J. Liemburg, Genetic Risk Outcome of Psychosis (GROUP) Investigators, Lieuwe de Haan, Frederike Schirmbeck, Claudia J. P. Simons, Therese van Amelsvoort, Wim Veling, Richard Bruggeman, Lisette van der Meer, Behrooz Z. Alizadeh

**Affiliations:** 1 Department of Epidemiology, University Medical Centre Groningen, University of Groningen, Hanzeplein 1, 9713 GZ Groningen, The Netherlands; 2 Department of Psychiatry, University Medical Centre Groningen, University Centre for Psychiatry, Rob Giel Research Centre, University of Groningen, Groningen, The Netherlands; 3 Department of Clinical and Developmental Neuropsychology, Faculty of Behavioural and Social Sciences, University of Groningen, Groningen, The Netherlands; 4 Department of Rehabilitation, Lentis Psychiatric Institute, Zuidlaren, The Netherlands

**Keywords:** Social inclusion, Social exclusion, Recovery, Schizophrenia, Psychosis, Cluster, Prediction

## Abstract

**Purpose:**

We aimed to explore the multidimensional nature of social inclusion (mSI) among patients diagnosed with schizophrenia spectrum disorder (SSD), and to identify the predictors of 3-year mSI and the mSI prediction using traditional and data-driven approaches.

**Methods:**

We used the baseline and 3-year follow-up data of 1119 patients from the Genetic Risk and Outcome in Psychosis (GROUP) cohort in the Netherlands. The outcome mSI was defined as clusters derived from combined analyses of thirteen subscales from the Social Functioning Scale and the brief version of World Health Organization Quality of Life questionnaires through K-means clustering. Prediction models were built through multinomial logistic regression (Model_MLR_) and random forest (Model_RF_), internally validated via bootstrapping and compared by accuracy and the discriminability of mSI subgroups.

**Results:**

We identified five mSI subgroups: “very low (social functioning)/very low (quality of life)” (8.58%), “low/low” (12.87%), “high/low” (49.24%), “medium/high” (18.05%), and “high/high” (11.26%). The mSI was robustly predicted by a genetic predisposition for SSD, premorbid adjustment, positive, negative, and depressive symptoms, number of met needs, and baseline satisfaction with the environment and social life. The Model_RF_ (61.61% [54.90%, 68.01%]; *P* =0.013) was cautiously considered outperform the Model_MLR_ (59.16% [55.75%, 62.58%]; *P* =0.994).

**Conclusion:**

We introduced and distinguished meaningful subgroups of mSI, which were modestly predictable from baseline clinical characteristics. A possibility for early prediction of mSI at the clinical stage may unlock the potential for faster and more impactful social support that is specifically tailored to the unique characteristics of the mSI subgroup to which a given patient belongs.

**Supplementary Information:**

The online version contains supplementary material available at 10.1007/s00127-024-02630-4.

## Introduction

Social inclusion is a fundamental human right that has posed serious challenges for patients with severe mental illness [1], constituting 5% of the global population [2]. Social inclusion has been described as “a multidimensional state where prevailing conditions enable full and active participation in all aspects of daily life” [3–5], where “multidimensional” is vaguely unrestricted to individual characteristics [6], relationships with other individuals and group environments [3, 6], subjective and objective living environments [7–10] and social-political rights [6]. Research on social inclusion is essential, as limited healthcare expenditures are spent on psychiatric disorder management (e.g., 0.47% of the Dutch total healthcare expenditures in 2017) [11–13], leaving problems other than clinical remission unattended. Such situation also applies to schizophrenia spectrum disorder (SSD) which accounts for one in five individuals with mental disorders [14, 15]. Guided SSD management elevates social inclusion, thus benefiting clinical and social recoveries, patients’ quality of life, and reducing burdens on healthcare organizations, and patient families. Therefore, investigating social inclusion for an indicative purpose, and identifying its predictive factors in SSD are crucial for promoting recoveries and preserving social inclusion in SSD.

Quantifying social inclusion and related concepts such as social capital, social participation and so forth [16] have faced challenges due to its multidimensional nature, ambiguous scope, conceptual complexity, lack of validated instruments and consequently absent application [16, 17]. We previously adopted a unidimensional construct of social inclusion through social functioning [18]. However, low social inclusion has been recognized in diversifying forms of low socio-economic status (e.g., unemployment, low education), a lack of functional recoveries, low social engagement, and quality of life (QoL), which have been studied as standalone conventional outcomes [19–22]. Therefore, as a broad holistic concept, social inclusion should be approximated by aggregating the aforementioned outcomes. In the present study, we expanded the uniform concept to multidimensional social inclusion by aggregating subscales of social functioning and additional QoL, to provide a more comprehensive evaluation of a patient’s social inclusion level. Hence, it remains to be studied whether patients’ subgroups of individuals experiencing similar mSI and the predictors of mSI subgroups are applicable.

Previous observational studies have identified factors and predictors of outcomes that are highly relevant to mSI, including socio-economic status (e.g., age, ethnicity, education, marriage status) [18, 21, 23, 24], genetic predisposition for SSD [18], early-life factors (e.g., premorbid adjustment and childhood trauma) [18, 24], disease profiles (e.g., types of diagnoses, cognition, symptoms, global functioning) [18, 21, 23–27], baseline work status [23], unmet needs [28], living environment [21, 29], and medication and substance use [18, 21]. Traditional approaches, such as regressions, have identified these (predictive) factors but have not been used in social inclusion prediction. In addition, as individual-level risk can maximize the utilities of prediction models and the intricate nature of mSI may benefit from a relaxed assumption of linearity, one solution is data-driven approaches. Due to their methodological advantages and focus on prediction accuracy, they have been increasingly applied in predicting SSD onset among ultra-high-risk populations [30, 31], psychosis outcomes including symptoms, treatment, relapsing and hospitalization [21, 32–36], and more recently, social aspects such as social recoveries, vocation, education and QoL [21, 36], with common algorithms such as support vector machine, decision tree and random forests. However, evidence has shown that their performance compared to standard approaches varies, depending on evaluation metrics relevant to the research question and/or clinical requirements [37–40]. Therefore, we hypothesized that mSI clusters exist within the SSD cohort. We also hypothesized that the data-driven approach might not perform worse and identify distinct predictors compared to the standard approach. We aimed to evaluate the predictability of mSI clusters by employing and comparing the performance of standard and data-driven approaches. We addressed firstly how many mSI subgroups can be identified in SSD. Secondly, what are the predictors of mSI, and which of the standard and data-driven approaches performs most accurately in predicting patients’ mSI at 3-year follow-up concerning model accuracy and discriminability.

## Methods

### Study design and population

**Fig. 1:**
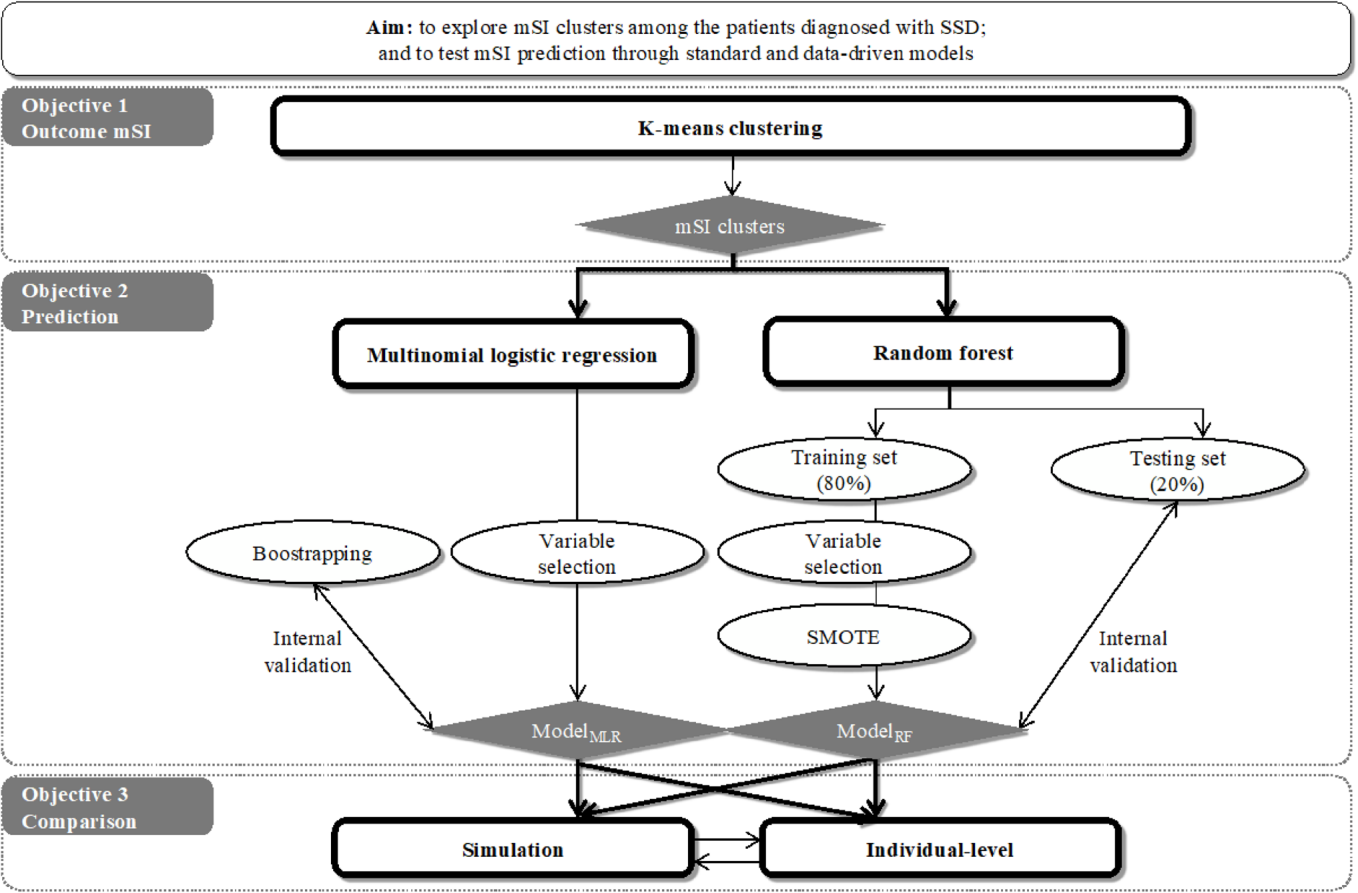
Study design and analytical framework. *SSD* schizophrenia spectrum disorder, *mSI* multidimensional social inclusion, *SMOTE* synthetic minority oversampling technique, *MLR* multinomial logistic regression, *RF* random forest

The overall study design is illustrated in Fig. 1. To identify subgroups of mSI, K-means clustering was applied. To predict mSI subgroups, prediction models were built using multinomial logistic regression (Model_MLR_) and random forest (Model_RF_), which were then internally validated. Lastly, to compare the two models, simulations and individual-level inspections were conducted.

We used the Dutch-nationwide database Genetic Risk and Outcome in Psychosis (GROUP) project, data release 8.0. Details on the GROUP project structure, participant recruitment, data collection and ethical approval have been published elsewhere [41]. In brief, 1119 patients were recruited at baseline across 36 partner mental health institutes including four university medical centres in Amsterdam, Groningen, Maastricht, and Utrecht. The inclusion criteria were: (1) age of 16–50 years; (2) fluent Dutch speaking; (3) diagnosis of a non-affective psychotic disorder according to DSM-IV; (4) the first contact with mental health care service no longer than 10 years ago; and (5) being able and willing to give written consent. The measurements taken at baseline and 3-year follow-up were used. Loss of follow-up was not applicable due to the naturalistic design of the GROUP project. As data was collected when a patient was visiting the clinics.

### Outcome

We defined mSI by the clusters derived from 13 subscales from two surveys: Social Functioning Scales (SFS) [42] and World Health Organization Quality of Life (WHOQOL-BREF) [43] (Supplementary Table S1), based on literature and data availability. These two validated well-developed self-reported questionaries were used to cover direct and indirect aspects of social inclusion in SSD. The baseline mSI was not available as SFS was not collected.

### Predictors

Potential predictors were included at baseline and/or 3-year follow-up based on literature, experts’ opinions and data availability. The inclusion of the 3-year follow-up in the analysis was mainly attributed to inspecting the predictability of a predictor at the baseline on the outcome mSI after the 3-year follow-up [44]. Covariables include domains of WHOQOL-BREF measured at baseline (physical, psychologic, social and environmental domains) [43]. The variables conceptually overlapping with the outcome mSI were considered predictors to avoid complicating and invalidating the mSI outcome conceptualized on the two validated questionnaires. For detailed predictors and explanations see Table 1.

**Table 1:** Characteristics of study population and predictors

Median [IQR]/*N* (%)	All patients	mSI subgroup					
		VLL	LL	HL	MH	HH	*P* value
	*N* = 1119 (100%)	*N* = 96 (8.58%)	*N* = 144 (12.87%)	*N* = 551 (49.24%)	*N* = 202 (18.05%)	*N* = 126 (11.26%)	
A. Model predictor^d^							
Medical centre							0.067
Amsterdam	283 (25.29)	24 (25.0)	46 (31.9)	130 (23.6)	52 (25.7)	31 (24.6)	
Groningen	287 (25.65)	30 (31.2)	40 (27.8)	134 (24.3)	58 (28.7)	25 (19.8)	
Maastricht	306 (27.35)	20 (20.8)	26 (18.1)	160 (29.0)	61 (30.2)	39 (31.0)	
Utrecht	243 (21.72)	22 (22.9)	32 (22.2)	127 (23.0)	31 (15.3)	31 (24.6)	
Age (year)	26.00 [22.00, 32.00]	29.00 [22.00, 34.25]	25.00 [21.00, 30.00]	25.00 [22.00, 32.00]	26.00 [21.00, 31.00]	27.00 [23.25, 31.00]	0.047
Gender: female	267 (23.86)	19 (19.8)	19 (13.2)	131 (23.8)	64 (31.7)	34 (27.0)	0.002
Ethnicity: non-Caucasian	256 (22.88)	27 (28.1)	28 (19.4)	155 (28.1)	25 (12.4)	21 (16.7)	< 0.001
PRS_SCZ_	− 3.16 [− 5.92, 0.00]	− 1.13 [− 4.82, 2.54]	− 3.49 [− 5.81, 0.25]	− 3.04 [− 5.90, − 0.05]	− 4.33 [− 7.04, − 1.71]	− 2.24 [− 5.54, 0.99]	< 0.001
PAS-overall	1.95 [1.42, 2.47]	2.15 [1.66, 2.74]	2.14 [1.72, 2.84]	1.95 [1.50, 2.44]	1.65 [1.16, 2.21]	1.82 [1.22, 2.30]	< 0.001
CTQ-total	1.53 [1.32, 1.80]	1.68 [1.36, 2.04]	1.55 [1.32, 1.80]	1.57 [1.39, 1.78]	1.34 [1.20, 1.60]	1.56 [1.30, 1.92]	< 0.001
IQ	94.00 [83.50, 105.00]	90.00 [83.00, 105.00]	94.00 [85.50, 103.00]	92.00 [82.00, 103.00]	100.00 [87.25, 110.00]	96.00 [87.00, 104.50]	< 0.001
Age of onset (year)	21.00 [18.00, 26.00]	23.00 [18.00, 29.00]	21.00 [18.00, 25.00]	21.00 [19.00, 27.00]	21.00 [18.00, 25.00]	22.00 [19.00, 26.00]	0.194
Duration of psychosis (year)	3.00 [1.00, 6.00]	3.50 [1.00, 8.00]	3.00 [1.00, 7.00]	3.00 [1.00, 6.00]	3.00 [1.00, 5.00]	4.00 [2.00, 7.00]	0.009
Diagnosis: affective	121 (10.81)	18 (18.8)	8 (5.6)	45 (8.2)	31 (15.3)	19 (15.1)	< 0.001
Positive symptoms	13.00 [9.00, 17.50]	15.00 [11.75, 21.00]	14.00 [11.00, 18.00]	14.00 [10.00, 18.00]	9.00 [7.00, 14.00]	13.00 [10.00, 17.75]	< 0.001
Core negative symptoms	10.00 [6.00, 13.00]	12.00 [7.75, 15.25]	11.00 [8.00, 15.00]	10.00 [7.00, 13.50]	6.50 [5.00, 9.00]	9.00 [6.00, 12.00]	< 0.001
Depressive symptom (frequency)	1.00 [0.62, 1.29]	1.26 [0.97, 1.75]	1.00 [0.75, 1.45]	0.96 [0.61, 1.25]	0.75 [0.50, 1.10]	1.12 [0.75, 1.52]	< 0.001
Depressive symptom (distress level)	1.40 [1.00, 1.82]	1.71 [1.39, 2.02]	1.41 [1.00, 1.75]	1.37 [1.00, 1.75]	1.23 [0.89, 1.76]	1.59 [1.14, 2.16]	< 0.001
GAF-disability	54.00 [42.00, 65.00]	47.50 [40.00, 55.00]	50.00 [41.00, 61.00]	51.00 [41.00, 65.00]	65.00 [50.25, 75.00]	55.00 [45.00, 65.00]	< 0.001
GAF-symptom	55.00 [45.00, 65.00]	50.50 [40.00, 60.00]	55.00 [40.00, 65.00]	55.00 [45.00, 65.00]	65.00 [55.00, 74.50]	60.00 [50.00, 65.00]	< 0.001
Remission (baseline to 3-year)^a^							< 0.001
No	365 (32.6)	63 (65.6)	80 (55.6)	144 (26.1)	22 (10.9)	56 (44.4)	
Yes (less than 6 months)	464 (41.5)	20 (20.8)	33 (22.9)	315 (57.2)	60 (29.7)	36 (28.6)	
Yes (over 6 months)	290 (25.9)	13 (13.5)	31 (21.5)	92 (16.7)	120 (59.4)	34 (27.0)	
Current urbanicity							0.005
No to little	422 (37.7)	39 (40.6)	72 (50.0)	191 (34.7)	75 (37.1)	45 (35.7)	
Moderate	238 (21.3)	11 (11.5)	26 (18.1)	137 (24.9)	36 (17.8)	28 (22.2)	
Strong to very strong	459 (41.0)	46 (47.9)	46 (31.9)	223 (40.5)	91 (45.0)	53 (42.1)	
Work: Full time or part time	615 (55.0)	46 (47.9)	77 (53.5)	297 (53.9)	125 (61.9)	70 (55.6)	0.183
Work payment							0.039
None	429 (38.34)	42 (43.8)	53 (36.8)	230 (41.7)	62 (30.7)	42 (33.3)	
Paid	404 (36.10)	31 (32.3)	48 (33.3)	194 (35.2)	91 (45.0)	40 (31.7)	
Voluntary	132 (11.80)	10 (10.4)	24 (16.7)	58 (10.5)	21 (10.4)	19 (15.1)	
Mixed	104 (9.29)	8 (8.3)	14 (9.7)	47 (8.5)	15 (7.4)	20 (15.9)	
Unknown	50 (4.47)	5 (5.2)	5 (3.5)	22 (4.0)	13 (6.4)	5 (4.0)	
Number of met needs	4.00 [2.00, 5.00]	4.00 [3.00, 6.00]	4.00 [3.00, 6.00]	4.00 [2.00, 5.00]	3.00 [1.00, 4.00]	4.00 [2.00, 6.00]	< 0.001
WHOQOL-BREF (baseline)							
Environment domain	3.57 [3.14, 3.99]	3.14 [2.71, 3.43]	3.43 [3.14, 3.86]	3.57 [3.14, 3.86]	4.00 [3.57, 4.30]	3.43 [3.00, 3.71]	< 0.001
Physical domain	3.43 [3.00, 3.86]	3.00 [2.52, 3.33]	3.43 [3.00, 3.71]	3.43 [3.00, 3.86]	3.86 [3.43, 4.29]	3.34 [2.86, 3.71]	< 0.001
Psychosocial domain	3.33 [2.83, 3.83]	2.77 [2.17, 3.17]	3.17 [2.83, 3.67]	3.33 [3.00, 3.67]	3.83 [3.33, 4.00]	3.17 [2.74, 3.50]	< 0.001
Social domain	3.33 [2.67, 3.67]	2.67 [2.33, 3.27]	3.00 [2.67, 3.67]	3.33 [2.67, 3.67]	3.67 [3.14, 4.00]	3.00 [2.67, 3.49]	< 0.001
Duration of using antipsychotics (year)	2.00 [0.00, 12.00]	3.50 [0.00, 22.00]	4.00 [0.00, 18.00]	3.00 [0.00, 12.00]	1.00 [0.00, 9.00]	2.00 [0.00, 10.75]	0.006
Chlorpromazine equivalents antipsychotic dose^b,c^							< 0.001
Drastic reduction	252 (22.52)	13 (13.5)	36 (25.0)	126 (22.9)	44 (21.8)	33 (26.2)	
Moderate reduction	308 (27.52)	25 (26.0)	31 (21.5)	159 (28.9)	65 (32.2)	28 (22.2)	
No change	114 (10.19)	13 (13.5)	19 (13.2)	27 (4.9)	35 (17.3)	20 (15.9)	
Moderate increase	202 (18.05)	14 (14.6)	24 (16.7)	113 (20.5)	34 (16.8)	17 (13.5)	
Drastic increase	243 (21.72)	31 (32.3)	34 (23.6)	126 (22.9)	24 (11.9)	28 (22.2)	
Awareness of antipsychotic use: (Become) aware^b,d^	1,003 (89.64)	79 (82.3)	125 (86.8)	523 (94.9)	163 (80.7)	113 (89.7)	< 0.001
Change of alcohol units consumed per week^b^	0.00 [− 2.00, 3.00]	0.00 [− 3.00, 0.25]	0.00 [− 1.25, 3.00]	1.00 [− 1.00, 3.00]	0.00 [− 2.00, 3.00]	0.00 [− 2.00, 1.00]	0.003
Change of cigarette units consumed^b^	0.00 [− 1.00, 4.00]	0.00 [− 2.50, 5.00]	0.00 [0.00, 3.00]	0.00 [− 1.00, 4.00]	0.00 [− 2.00, 0.00]	0.00 [0.00, 3.00]	0.01
Change of total week with cannabis consumption in past 12 months^b^	0.00 [0.00, 2.00]	0.00 [0.00, 0.00]	0.00 [− 0.25, 0.00]	0.00 [− 0.50, 4.50]	0.00 [0.00, 0.00]	0.00 [0.00, 0.00]	< 0.001

*VLL* “very low/very low” mSI subgroup characterized by the lowest levels of social functioning and quality of life while the quality of life is even worse, *LL* “low/low” mSI subgroup featured by low levels of social functioning and quality of life but moderately better quality of life, *HL* “high/low” mSI subgroup with a high social functioning but low quality of life, *MH* “medium/high” mSI subgroup with a medium level social functioning but a relatively high level of quality of life, *HH* “high/high” mSI subgroup featured by the highest level of both social functioning and quality of life

^a^Remission is a longitudinal variable that recorded a patient’s remission status between the baseline and 3-year follow-up

^b^The variable indicates change from baseline to 3-year follow-up. For the continuous variables, a positive, negative and 0 value implies an increased, decreased and no change in use

^c^Specifically, the antipsychotic dose excluding no dose change (0 value) were divided by 25 percentiles into four groups which were denoted as drastic reduction, moderate reduction, moderate increase, and drastic increase

^d^Being (or becoming) aware of antipsychotic use refers to the patients who are aware of them using antipsychotic at baseline and 3-year follow-up (or being unaware at the baseline and be)

^e^For explanation: a higher value of PRS_SCZ_ (polygenic risk score for schizophrenia) indicates a stronger genetic predisposition of developing SSD (schizophrenia spectrum disorder); PAS (Premorbid Adjustment Score) has a 7-point scale for each item in different age periods (0 healthiest adjustment, 6 lowest adjustment) and PAS-overall is an average score retrospectively measured for childhood (before age 12), early adolescence (12–16) and late adolescence1(6–19) prior to the disease onset where a higher PAS-overall reflects a worse premorbid adjustment [85]; CTQ (Childhood Trauma Questionnaire, Dutch Version) has a 5-point scale for each item (1 never true, 5 very often true) in each subscale (i.e., emotional abuse, physical abuse, sexual abuse, emotional neglect and physical neglect) and CTQ-total is an average score of all subscales where a higher CTQ-total reflect a severer childhood adversity [86]; a higher IQ indicates a better cognitive ability; a higher value of positive symptoms or core negative symptoms (i.e., Blunted affect, Emotional withdrawal, Poor rapport, Lack of spontaneity, Active social avoidance) from PANSS (Positive And Negative Syndrome Scale) indicates severer symptoms [87]; depressive symptoms (frequency and distress level) from CAPE (community assessment of psychic experience) have a 4-point scale (1 never, 4 nearly always; 1 not distressed, 4 very distressed) [88]; a higher value of GAF (Global Assessment of Functioning)-disability or -symptoms indicates less severe disability or symptoms (i.e., less impairment) [89]; from Camberwell Assessment of Need (CAN), a higher number of met needs suggests a better-fulfilled individual needs [90]; four domains of WHOQOL-BREF (the abbreviated version of the World Health Organization Quality of Life) has a 5-point scale for each domain item, and each domain score ranges from 1 (very dissatisfied) to 5 (very satisfied) [43]

### Data analysis and statistical modeling

To construct outcome mSI, all subscales of SFS and WHOQOL-BREF were standardized and used to cluster patients with K-means clustering (Fig. 1, Objective 1). The assumption of K, symbolizing the number of subgroups, was premised on statistical indexes (i.e., silhouette, duda, pseudot2, Hartigan and gap indexes with the Euclidean distance) [45] and supplemented with clinical knowledge. Subgroup (between clusters) difference was assessed by Kruskal–Wallis tests. The two-group comparison was examined by Dunn’s Kruskal–Wallis Multiple Comparison tests and Bonferroni adjustment.

Prediction models were parallelly constructed via multinomial logistic regression (Model_MLR_, standard approach) and random forest (Model_RF_, data-driven approach) (Fig. 1, Objective 2). In the Model_MLR_, the subgroup with the best mSI level was chosen as the reference group because of our particular interest in the groups with relatively worse mSI levels under the probable mimicry of the subgroup with the best mSI to healthy controls. We presented odds ratios (ORs), confidence interval (95% CI), model performance including accuracy (i.e., 1-misclassification rate) and its 95% CI derived from bootstrapping. As for the Model_RF_, we reported the variable importance of the identified predictors (i.e., derived from a variable-specific out-of-bag decrease in accuracy averaged over all trees after permutation), where a higher value indicates higher usefulness of a variable in prediction. We also reported the model performance metric, including accuracy in both training and testing and the *P* value of the one-sided binomial test. In addition, considering the imbalanced outcome, *P* values of the one-sided binomial test in both models were reported to examine if the model accuracy is significantly better than no information rate (NIR), suggesting if the model can allocate a patient into the right outcome group significantly better than classification by chance. Finally, complete-case sensitivity analyses were conducted and reported in Supplementary Results.

Two models were compared by accuracy and mSI-cluster discriminability (Fig. 1, Objective 3). We conducted simulations with 1000 repeats using a random draw of 30%, 50%, 70%, 80% and 90% of the total sample. Furthermore, the individual-level prediction accuracy and mSI-cluster discriminability of both models were also examined by scatterplots and confusion matrix. Such evaluation shares similarities with the use of the Area Under the Curve (AUC), which is typically employed for binary outcomes.

The data analyses were conducted using R version 1.4.1103[46]. Technical details including outlier inspection, missingness and imputation, statistical power, justification of the chosen algorithm and model constructions were illustrated in Supplementary Methods.

## Results

### Clusters of multidimensional social inclusion

**Fig. 2:**
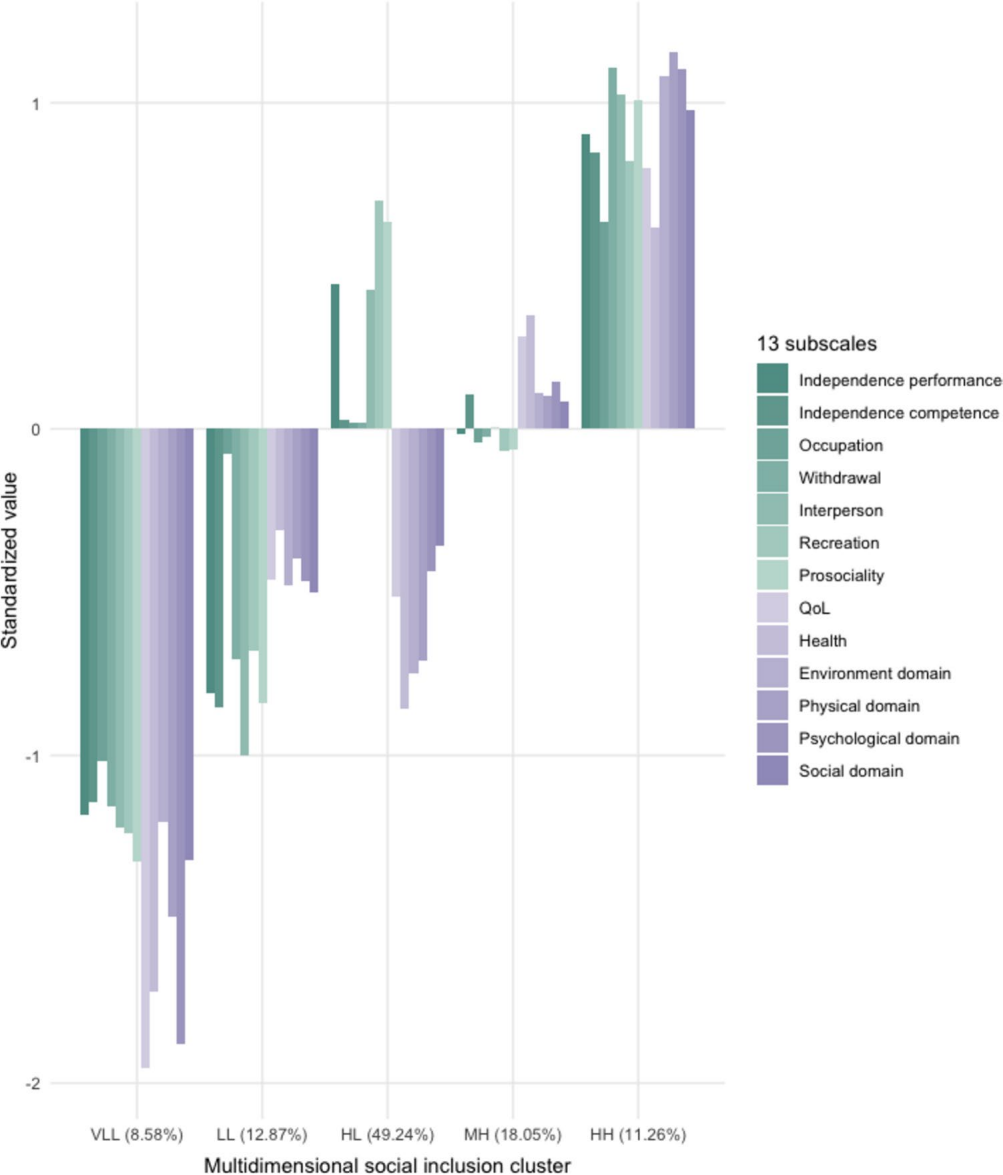
Centroid feature of subgroups of multidimensional social inclusion. Green color represents the seven subscales from SFS (Social Functioning Scale), and purple color represents the six subscales from WHOQOL-BREF (the abbreviated version of World Health Organization Quality of Life). *VLL* “very low/very low” mSI subgroup characterized by the lowest levels of social functioning and quality of life while the quality of life is even worse, *LL* “low/low” mSI subgroup featured by low levels of social functioning and quality of life but moderately better quality of life, *HL* “high/low” mSI subgroup with a high social functioning but low quality of life, *MH* “medium/high” mSI subgroup with a medium level social functioning but a relatively high level of quality of life, *HH* “high/high” mSI subgroup featured by the highest level of both social functioning and quality of life

Figure 2 demonstrates the centroid features of five subgroups identified: (1) “**very low** (social functioning)**/very low** (QoL)” cluster (VLL, 8.58% of patients); (2) “**low/low**” cluster (LL, 12.87%); (3) “**high/low**” cluster (HL, 49.24%); (4) “**medium/high**” cluster (MH, 18.05%); and (5) “**high/high**” cluster (HH, 11.26%) (Statistical indexes see Supplementary Table S2). Differences in 13 subscales among 5 groups were observed (Supplementary Table S3). To gain statistical power, we merged VLL and LL due to their similarities in low social functioning, ending up with four multinomial subgroups: “low/low” (LL, 21.45%), “high/low” (HL, 49.24%), “medium/high” (MH, 18.05%), and “high/high” (HH, 11.26%). Patient characteristics of all included patients and patients in each mSI cluster are shown in Table 1.

### Multinomial logistic regression model

The Model_MLR_ included 22 predictors (Table 2), among which PAS (LL vs HH, 1.66 [1.22, 2.26]; HL vs HH, 1.43 [1.07, 1.92]), PRS_SCZ_ (HL, 0.95 [0.91, 0.99]; MH, 0.93 [0.88, 0.98]), presence of positive (MH, 0.91 [0.86, 0.96]), core negative symptoms (LL, 1.08 [1.02, 1.15]; HL, 1.07 [1.01, 1.13]), and frequency of depressive symptoms (MH, 0.58 [0.37, 0.91]), symptomatic remission (MH, 3.19 [1.90, 5.36]; HL, 0.37 [0.26, 0.54]), number of met needs (MH, 0.86 [0.78, 0.95]), baseline WHOQOL-BREF environment and social domains (MH, 3.09 [1.79, 5.35] and 1.55 [1.06, 2.27] respectively) were identified as some of the more important predictors. Overall, the model accuracy was 59.16% (bootstrapping 95% CI [55.75%, 62.58%]; *P* = 0.994).

**Table 2:** Effects (odds ratios) of predictors estimated by the multinomial logistic regression model

Predictor	Outcome				Predictor	Outcome			
	LL	HL	MH	HH (REF)		LL	HL	MH	HH (REF)
	*N* = 240	*N* = 551	*N* = 202	*N* = 126		*N* = 240	*N* = 551	*N* = 202	*N* = 126
	OR [95%CI]	OR [95%CI]	OR [95%CI]			OR [95%CI]	OR [95%CI]	OR [95%CI]	
A. Socio-demography					D. Work, social and overall environment				
Medical centre					Current urbanicity				
Amsterdam	REF	REF	REF	1.00	No to little	REF	REF	REF	1.00
Groningen	1.47 [0.73, 2.95]	1.72 [0.90, 3.32]	1.56 [0.73, 3.34]		Moderate	0.79 [0.54, 1.17]	1.09 [0.76, 1.57]	1.00 [0.65, 1.54]	
Maastricht	0.83 [0.41, 1.64]	1.49 [0.80, 2.77]	1.05 [0.50, 2.18]		Strong to very strong	1.71 [1.07, 2.76]*	1.09 [0.72, 1.65]	1.25 [0.74, 2.09]	
Utrecht	0.85 [0.43, 1.68]	1.15 [0.61, 2.17]	1.06 [0.48, 2.35]		Work				
Age	1.01 [0.98, 1.05]	1.02 [0.98, 1.05]	0.98 [0.94, 1.02]		Not working or unknown	REF	REF	REF	1.00
Gender					Full time or part time	1.55 [0.56, 4.35]	3.00 [1.13, 7.99]*	1.34 [0.40, 4.55]	
Male	REF	REF	REF	1.00	Work payment				
Female	0.53 [0.29, 0.97]**	0.74 [0.44, 1.25]	1.00 [0.54, 1.85]		None	REF	REF	REF	1.00
Ethnicity					Paid	0.63 [0.20, 1.97]	0.33 [0.11, 0.98]*	1.35 [0.36, 5.10]	
Caucasian	REF	REF	REF	1.00	Voluntary	0.61 [0.27, 1.39]	0.40 [0.19, 0.88]*	0.98 [0.38, 2.54]	
Non-Caucasian	1.56 [0.80, 3.06]	2.22 [1.20, 4.12]*	1.32 [0.61, 2.86]		Mixed	0.40 [0.12, 1.36]	0.24 [0.08, 0.77]*	0.57 [0.13, 2.47]	
B. Gene and early-life					Unknown	0.55 [0.11, 2.65]	0.41 [0.10, 1.77]	2.19 [0.38, 12.57]	
PAS-overall	1.66 [1.22, 2.26]**	1.43 [1.07, 1.92]*	1.31 [0.92, 1.85]		Number of met needs	1.02 [0.93, 1.10]	0.94 [0.87, 1.02]	0.86 [0.78, 0.95]**	
PRS_SCZ_	0.98 [0.93, 1.03]	0.95 [0.91, 0.99]**	0.93 [0.88, 0.98]**		WHOQOL-BREF-environment	1.03 [0.65, 1.65]	1.35 [0.87, 2.09]	3.09 [1.79, 5.35]**	
C. Disease profile					WHOQOL-BREF-social	1.12 [0.80, 1.57]	1.32 [0.97, 1.80]	1.55 [1.06, 2.27]*	
Diagnosis					E. Medication use				
Non-affective	REF	REF	REF	1.00	Duration of using antipsychotics	1.01 [1.00, 1.02]*	1.01 [1.00, 1.02]	1.00 [0.99, 1.01]	
Affective	0.80 [0.40, 1.58]	0.62 [0.33, 1.14]	1.26 [0.62, 2.54]		Antipsychotic dose (in Chlorpromazine equivalents)				
Duration of psychosis	0.95 [0.88, 1.03]	0.94 [0.87, 1.00]	0.97 [0.89, 1.06]		Drastic reduction	REF	REF	REF	1.00
Positive symptoms	0.98 [0.94, 1.02]	0.98 [0.94, 1.02]	0.91 [0.86, 0.96]**		Moderate reduction	1.47 [0.75, 2.88]	1.87 [1.03, 3.41]*	2.21 [1.08, 4.50]*	
Core negative symptoms	1.08 [1.02,1.15]**	1.07 [1.01, 1.13]*	0.97 [0.91, 1.05]		No change	1.34 [0.61, 2.97]	0.49 [0.23, 1.05]	1.58 [0.68, 3.66]	
Depressive symptom (frequency)	1.14 [0.71, 1.83]	0.58 [0.37, 0.91]**	0.65 [0.37, 1.13]		Moderate increase	1.47 [0.67, 3.21]	2.57 [1.27, 5.17]**	2.29 [0.99, 5.30]	
GAF-symptom	0.99 [0.97, 1.01]	0.98 [0.96, 1.00]**	0.96 [0.94, 0.99]**		Drastic increase	1.42 [0.71, 2.84]	1.81 [0.96, 3.41]	0.98 [0.43, 2.23]	
Remission (baseline to year 3)					Awareness of antipsychotic use				
No	REF	REF	REF	1.00	No	REF	REF	REF	1.00
Yes (≤ 6 months)	0.68 [0.44, 1.06]	1.11 [0.73, 1.68]	3.19 [1.91, 5.36]**		(Become) aware	0.69 [0.33, 1.45]	2.89 [1.38, 6.06]**	0.65 [0.30, 1.41]	
Yes (> 6 months)	1.04 [0.67, 1.61]	0.37 [0.26, 0.54]**	0.66 [0.42, 1.05]		Constant	0.31 [0.02, 5.57]	0.19 [0.01, 2.79]	0.23 [0.01, 6.53]	
F. Model performance									
AIC	2336.84 [2232.61, 2441.08]				Accuracy	59.16% [55.75%, 62.58%]			
Log Likelihood	− 1069.42 [− 1121.54, − 1017.30]				* P* value [Accuracy > NIR]	0.994			
McFadden pseudo R^2^	0.23 [0.19, 0.26]				Kappa	0.34 [0.28, 0.40]			
Adjusted McFadden pseudo R^2^	0.21 [0.18, 0.24]								

*REF* reference group, *LL* mSI subgroup with both low social functioning and quality of life, *HL* mSI subgroup with a high social functioning but low quality of life, *MH* mSI subgroup with a medium level social functioning but a relatively high level of quality of life, *HH* mSI subgroup featured by the highest level of both social functioning and quality of life, *PAS* Premorbid Adjustment Score, *PRS*_*SCZ*_ polygenic risk score for schizophrenia, *GAF* Global Assessment of Functioning, *WHOQOL-BREF* the abbreviated version of World Health Organization Quality of Life, *NIR* no information rate

The significance level is represented by *P < 0.05; **P < 0.01; ***P < 0.001. 95%CI of model performance were derived from bootstrapping

### Random forest model

**Fig. 3:**
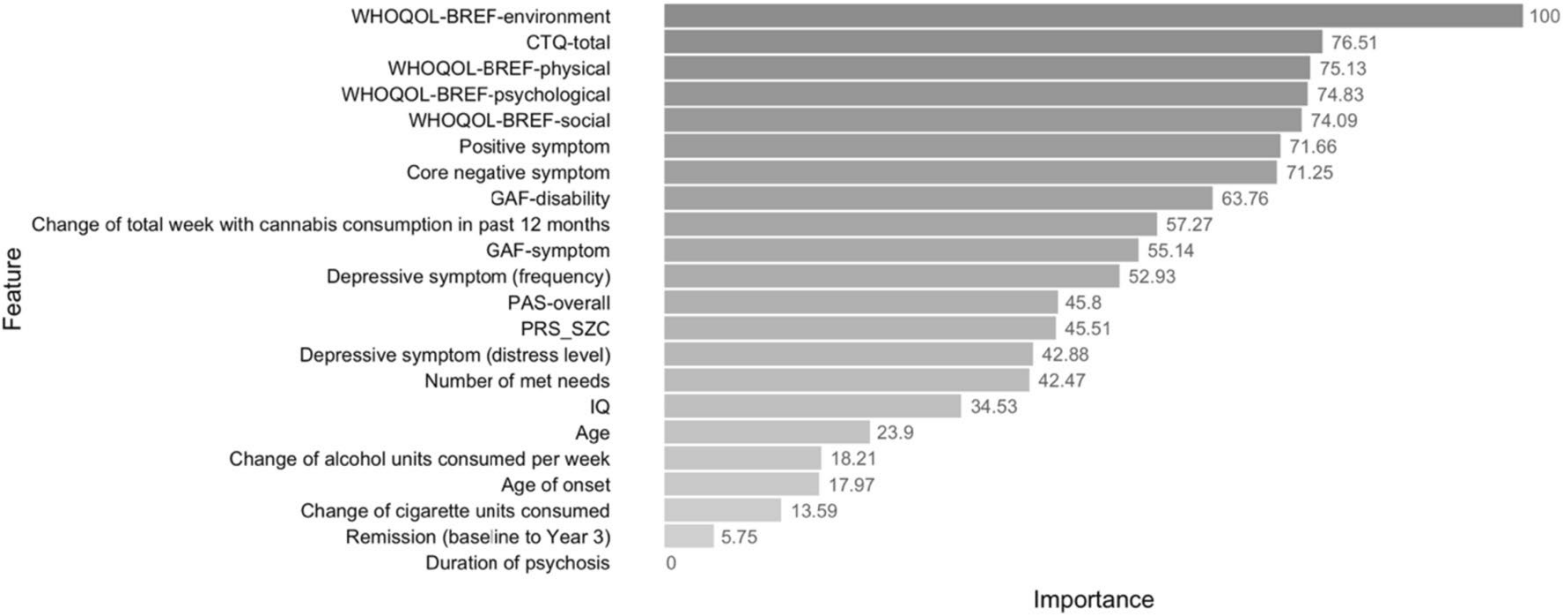
Variable importance provided by the random forest model*.* *WHOQOL-BREF* the abbreviated version of World Health Organization Quality of Life, *CTQ* Childhood Trauma Questionnaire, Dutch Version, *GAF* Global Assessment of Functioning, *PAS* Premorbid Adjustment Score, *PRS*_*SCZ*_ polygenic risk score for schizophrenia

The Model_RF_ identified 22 predictors, among which WHOQOL-BREF domain scores and CTQ-total contributed the most to predicting the mSI group (Fig. 3). Observing the feature importance indices, other important factors were age, PRS_SCZ_, PAS-overall, symptom severity (positive, core negative and depressive) and the number of met needs. The accuracies on training and testing sets, respectively, were 70.46% ± 2.03% and 61.61% (95% CI [54.90%, 68.01%]; *P* =0.013).

### Model comparison

**Fig. 4:**
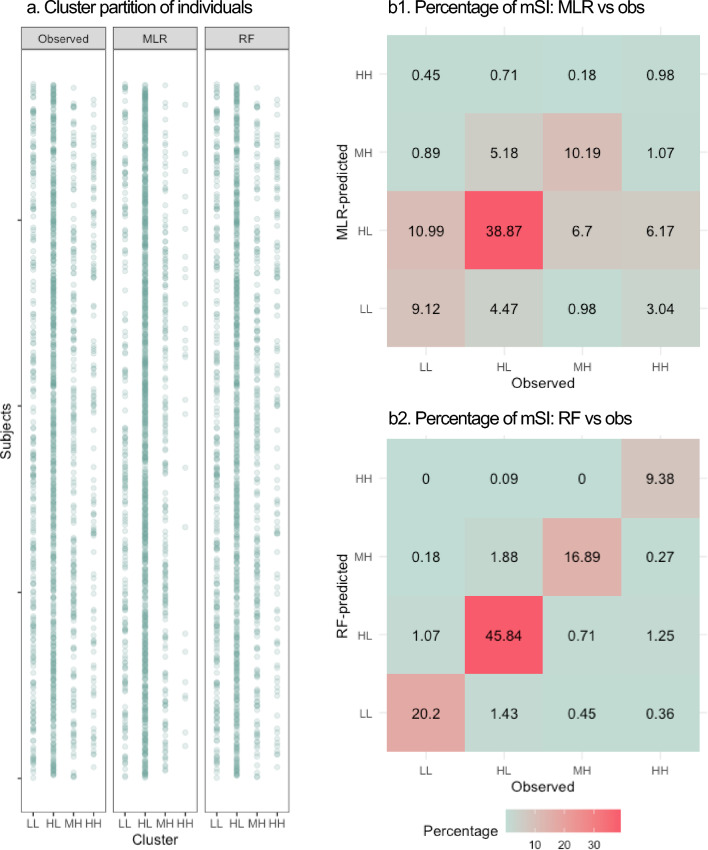
Individual level of prediction results. The figure used the complete data of 1119 patients to assess the model on an individual level. **a** plotted pattern overview from the observed, MLR-predicted, and RF-predicted mSI clusters. **b** demonstrated the overall model accuracy (i.e., secondary diagonal sum) and the percentage of each mSI cluster which was correctly and incorrectly predicted by the Model_MLR_ and Mdoel_RF_ compared to the observations. From the figure below, we observed comparable distributions between the observed and RF-predicted mSI clusters. Dissimilarly, the MLR-predicted mSI clusters displayed a higher proportion in HL in trade of apparent lower percentages in LL and HH. *LL* mSI subgroup with both low social functioning and quality of life, *HL* mSI subgroup with high social functioning and low quality of life, *MH* mSI subgroup with medium social functioning and high quality of life, *HH* mSI subgroup with both high social functioning and quality of life, *MLR* multinomial logistic regression, *RF* random forest, *obs* observations

For the simulation of 30% (up to 90%) of the observed patients, the mean accuracy of Model_MLR_ was 59.12% ± 2.50% (59.13% ± 1.20%) and the mean of Model_RF_ was 92.29% ± 1.34% (92.26% ± 0.67%; Supplementary Table S4). In the comparison of observed and predicted mSI subgroups among 1119 patients, 662 patients (59.16%) and 1033 patients (92.31%) were correctly predicted by Model_MLR_ and Model_RF_ correspondingly. We saw a similar pattern and percentage of mSI subgroup partition between the observations and RF-predictions, and contrarily, differences occurred between the observations and MLR-predictions (Fig. 4a). For example, a distinctly higher HL percentage with a (62.73% vs 49.24% observed, 48.88% RF-predicted, Fig. 4b).

## Discussion

We identified five mSI subgroups, including VLL, LL, HL, MH and HH. The Model_MLR_ and Model_RF_ consistently regard PRS_SCZ_, premorbid adjustment, symptoms, baseline environment, social domains and numbers of met needs as paramount predictors for mSI at 3-year follow-up. Comparatively, the Model_RF_ is cautiously considered better for its discriminability of all mSI subgroups. The mSI clusters intend to preliminarily define and bring awareness to social inclusion, an understudied but crucial outcome in SSD. The mSI prediction models should be further improved and externally validated for possible profound clinical and policy implications.

### Clusters of multidimensional social inclusion among patients with schizophrenia spectrum disorder

Among the five mSI subgroups, the “very low (social functioning)/very low (QoL)” (VLL) and “low/low” (LL) indicated a low mSI level. VLL demonstrated the lowest social functioning and QoL in the spectrum with an even worse QoL, while LL exhibited relatively low levels of social functioning and QoL but slightly higher QoL. The VLL and LL combined, totalling 21.45%, presented more prominent core negative symptoms and worse premorbid adjustment compared to other mSI subgroups. Given that approximately 80% of the participants were diagnosed with schizophrenia [47] and a recent meta-analysis has indicated a 32.19% global prevalence in deficit schizophrenia [48], characterized by primary and persistent negative symptoms that distinguish it from non-deficit schizophrenia [49], the amalgamated group might align with deficit schizophrenia. Furthermore, VLL and LL were merged for the analysis, yet disparities exist. Patients in VLL, compared to LL, were more likely to be non-Caucasian, genetically vulnerable for SSD, have affective psychosis, depressed, have lower IQ, have higher levels of childhood trauma and symptoms, and showed less remission. This may eventually lead to restricted access to the labor market, recreational activities, and social engagement with simultaneously affected QoL and mSI.

The “high/low” (HL) and “medium/high” (MH) implied a medium mSI level. Compared to LL, HL showed a better level of social functioning, particularly in the areas of independence performance, interperson, recreation and prosociality. This improvement could be attributed to their higher premorbid adjustment, slightly less severe symptoms (especially core negative symptoms), and higher rates of symptomatic remissions. However, LL and HL had similar levels of QoL (moderately lower than the average QoL of all patients with SSD), with HL displaying a distinctively lower satisfaction toward general health, environment, and physical conditions. The disparities between LL and HL might suggest that the patients in HL probably experience increased social exposure through recreational and social activities along with psychologic impacts such as internalized stigma, and a systematically perceived low QoL [50]. Differently, MH exhibited an average social functioning level and mildly higher QoL than the average QoL of all patients with SSD, which could be resulted from better IQ, less severe symptoms, stable remission over six months and fewer met needs. No significant difference in occupation was observed among LL, HL and MH which corroborates with previous studies [51, 52]. Contrarily, HL and MH with better function were likely to stay at a job. The final mSI subgroup, “high/high” (HH), showed a high mSI level with the highest levels of social functioning and QoL, suggesting that this subgroup mimics healthy controls, albeit on an overall lower-level contrary to healthy controls. Notably, patients with medium-to-high mSI (HL, MH and HH) demonstrated fewer core negative symptoms, better premorbid adjustment and a higher rate of symptomatic remission over six months. These characteristics could correspond to diagnostic categories such as acute and transient psychotic disorders (ATPD) and non-affective acute remitting psychosis (NARP), marked by abrupt onset of psychotic symptoms within two weeks and early, complete remission [53–55].

Therefore, mSI is essential, as a holistic approach, to provide a comprehensive overview of social inclusion of an individual. The subgroup characteristics could also guide intervention strategies. Patients in VLL and LL may require more psychosocial interventions to manage symptoms and improve social functioning. Priority should be given to their eligibility for protected living when independent living is not achievable. While patients in HL could be targeted with current programs aiming for long-term remission and stigma reduction, patients in MH and HH could benefit from training for advanced skills and opportunities for more challenging job positions, which enhance self-esteem and self-actualization, ultimately improving mSI.

### Common factors predictive of multidimensional social inclusion

The Model_MLR_ identified predictors such as gender, ethnicity, current urbanicity, antipsychotic dose, among others. In contrast, the Model_RF_ selected predictors including baseline all QoL domains, childhood adversity, and change of total week with cannabis consumption in the past 12 months among others. Noteworthily, eight important common predictors were shared between both models. Congruent with previous studies [18, 56–59], in Model_MLR_, more severe core negative symptoms increased the risk of having low-to-medium mSI (LL and HL relative to HH). Premorbid adjustment had the highest negative effect on low-to-medium mSI in Model_MLR_ (LL and HL relative to HH). Thus, premorbid adjustment showed moderate predictability of the 3-year mSI. This is aligned with earlier studies which have shown that worse premorbid adjustment may lead to poorer social outcomes later in the course of SSD [60–64]. Therefore, premorbid adjustment is undoubtfully vital and can be potentially used for screening of low mSI. Yet, it has not caught enough attention in the field given the limited literature.

Surprisingly, worse positive symptoms significantly predicted good mSI (i.e., MH relative to HH) with a mild protective effect. Other studies have found that positive symptoms do not contribute much to QoL or social cognition [65–67], although the cross-sectional symptomatic remission [68] can significantly improve social functioning [69]. A higher genetic vulnerability toward SSD displayed a significant protective effect on good mSI (HL and MH relative to HH). This is possibly due to the single comparison in a relatively low sample size of HH in a multivariate model, which may occupy the variability of mSI concerning PRS_SCZ_, and consequently yielded a dubious finding. Therefore, the relationship between genetic predisposition and mSI should be independently investigated in well-powered research. Counterintuitively, we found that the more often an SSD patient experienced depressive symptoms, the more likely the patient was to be in high mSI (HH) than in medium mSI (HL) in the Model_MLR_, which is discordant with previous studies [70–72]. One possible explanation is that in the diagnostic categories of SSD, patients with a higher level of depressive symptoms are more likely to have affective symptoms and to be associated with affective dysregulation, which results in a better outcome (mSI in our case) than the one of the patients with non-affective symptoms such as withdrawal in HL [73]. Specifically, the early detection and interventions of depressive symptoms could be essential to help patients with SSD, which might further impact their lives and subsequent mSI. All the aforesaid factors were confirmed informative in the Model_RF_ as well.

Abundant studies have emphasized the importance of occupation (and thus financial income and opportunities for acquiring new skills) and social relationships [74, 75], secure stable housing, family support [76] and inclusive and accessible support systems across sectors such as transportation [76, 77] for social inclusion. On the other hand, a higher level of fulfilled needs significantly distinguished MH and HH only in Model_MLR_. Particularly in the Model_RF_, the number of met needs was considered necessary. Therefore, with a growing emphasis on extramural care [78], the local communities and mental health organizations need to incorporate the heterogeneous environmental and social needs, beyond the medical needs, of patients with SSD at different mSI levels.

In contrast to the univariable analysis, we identified the duration of psychosis as a predictor with limited contribution in the Model_MLR_ or even with zero importance in the Model_RF_ for predicting mSI. This might be influenced by the presence of the other important predictors exemplified by QoL-related factors and childhood trauma (Fig. 3). The exclusion of variables related to duration of psychosis such as age at baseline and age of onset from the Model_RF_ did not improve the low importance of duration of psychosis. While previous studies have suggested an association between a shorter duration of psychosis and favorable changes in symptomatic remission and social functioning [79, 80], limited and conflicting evidence hinders confirmation of the association between chronicity and symptom severity, functioning and QoL [80, 81]. Therefore, the duration of psychosis might be less relevant, resulting in a modest contribution to predicting mSI at 3 years in multivariable models.

Despite many common factors that were selected in the both models, PRS_SCZ_, positive, negative, depressive symptoms, premorbid adjustment, baseline environment and social-domain satisfactions and the number of met needs were found to be crucially predictive of mSI.

### Model performance in prediction

The predictivities of Model_MLR_ and Model_RF_ were fair and comparable. We inferred the Model_RF_ outperforms as it allocated individuals to the correct mSI cluster significantly better than chance alone, suggesting caution in applying the Model_MLR_ as some predictors in the Model_MLR_ were indiscriminative to mSI subgroups except for the HL cluster due to the imbalanced mSI outcome. Furthermore, although Model_RF_ did not perform as well as expected, earlier studies using data-driven methods have reported similar accuracies of 60%-75%, highlighting the complexity of SSD. The holistic mSI measure (compared to a single clinical outcome) and a longer time interval of 3-year (compared to 1 year) may be influenced vastly by various factors and their interactions, making mSI prediction challenging. To be integrated into clinical practices through electronic health records (EHR) (Supplementary Clinical Illustration) [82], the Model_RF_ requires external validation through international data-sharing efforts [83]. Despite its replicability, the model implementation may encounter a prolonged journey. Aligned with the minority of readily implementable psychiatry prediction models, the Model_RF_ requires inputs easily obtainable in clinical settings [84]. However, addressing the challenge of accurately and efficiently computing PRS_SCZ_ from available and affordable genotype data remains essential, especially when considering the opportunity costs of assisting individuals facing poor social inclusion [83].

### Future perspectives

Future studies should continue working on the conceptualization of mSI and examining its applicability across diagnoses. Meanwhile, developing a validated composite score could enable longitudinal monitoring. Methodologically, future studies should take extra steps in modeling procedures such as outer cross-validation and different feature-selection algorithms and give opportunities to the latest interpretable machine learning algorithms for exertion to pick up next-level clinical utility. Clinically, when building prediction models, future studies should test the utilities of potential factors that measure similar clinical outcomes but with slight variations in submodels and understand the multidimensional mechanisms hidden under the effect sizes such as premorbid adjustment. Furthermore, investigations on the genetic effects on behaviors and mSI along the course of SSD could be necessary for early screening. Statistically, other observable and non-observable factors, such as personality traits, coping strategies, diversity of community residents, community social-economic status, relationship with caregivers, and so forth, might simultaneously be more essential for improving the mSI prediction. With growing awareness of social inclusion and the development of sophisticated prediction models for mSI, along with personalized interventions and supporting policies, patients with SSD would be able to acquire necessary skills and receive essential resources. This would consequently aid them in managing their conditions and achieving great inclusion in society.

### Study strengths and limitations

We quantified the multidimensional nature of social inclusion by combining thirteen subscales of the self-reported SFS and WHOQOL-BREF without the intention of developing a validated questionnaire. Our conceptualization emphasizes the multidimensionality that provides a comprehensive overview of an individual’s status of social inclusion through a broad range of activities, the perception of an individual, and the exploitation of the existing large cohort and standardly collected data. The longitudinal measurements in the cohort were utilized. We also compared standard and data-driven models to examine the robustness and enhance the credibility of the factors and predictability of mSI. However, no baseline mSI was available to investigate mSI changes over time. The models require external validation and could be improved with more non-clinical data.

Despite previous efforts in developing conceptual frameworks for social inclusion, it is vital to acknowledge that the constitution of social inclusion is nebulous, implying a variable boundary of this multifaceted construct. The available data do not provide much flexibility or balance the eligibility and validity of the elements used for the construct. No interview was conducted to preliminarily determine the most relevant scope of social inclusion.

## Conclusion

We introduced mSI which is backboned by social functioning and quality of life, resulting in five identified subgroups including “very low/very low”, “low/low”, “high/low”, “medium/high”, and “high/high”. We found that genetic predisposition for SSD, premorbid adjustment, positive, negative and depressive symptoms, number of met needs and baseline satisfaction with the environment and social life were robust factors predictive of mSI in SSD. We cautiously concluded that the Model_RF_ offered a better prediction, compared to the Model_MLR_, of the 3-year mSI among patients with SSD due to its better discriminability. Yet, continuous model refinement and external validation are still required.

Our findings indicate that mSI is applicable and offers possibilities for personalized treatment strategies and policymaking tailored for patients with SSD at different mSI levels. Our study emphasizes the special proposition of mSI as an imperative goal for patients with SSD and a possible solution to expensive healthcare and societal harmony.

## Supplementary Information

Below is the link to the electronic supplementary material.

Supplementary file1 (DOCX 357 kb)

## Declarations

### Availability of data and material

Due to privacy, we are not permitted to share the data. Any researcher interested in acquiring the dataset can submit an application form to the GROUP committee via e-mail.
