# Supplementary material for: Shaping tomorrow’s support: baseline clinical characteristics predict later social functioning and quality of life in schizophrenia spectrum disorder
Source: Soc Psychiatry Psychiatr Epidemiol. 2024 Mar 8;59(10):1733–50. doi: 10.1007/s00127-024-02630-4 (PMC11464570; doi:10.1007/s00127-024-02630-4)
Supplement: Supplementary file 1 — Supplementary file1 (DOCX 357 kb) [file 127_2024_2630_MOESM1_ESM.docx]

# SUPPLEMENTARY INFORMATION

**Shaping tomorrow’s support: baseline clinical characteristics predict later social functioning and quality of life in schizophrenia spectrum disorder**

J Hao *et al*.

Content

[SUPPLEMENTARY METHODS 2](#_Toc160533063)

[Outliers, missingness, and imputation 2](#_Toc160533064)

[Sample size and power of the study 2](#_Toc160533065)

[Prediction modelling 2](#_Toc160533066)

[SUPPLEMENTARY RESULTS 4](#_Toc160533067)

[Complete-case analyses 4](#_Toc160533068)

[SUPPLEMENTARY CLINICAL ILLUSTRATION 5](#_Toc160533069)

[A real case example 5](#_Toc160533070)

[SUPPLEMENTARY TABLES 6](#_Toc160533071)

**[Table S1](#_Toc160533072)** [Thirteen subscales used for constructing outcome multidimensional social inclusion 6](#_Toc160533072)

**[Table S2](#_Toc160533073)** [Statistical indexes of K-means clustering 7](#_Toc160533073)

**[Table S3](#_Toc160533074)** [Subgroup differences in multidimensional social inclusion by 13 subscales 8](#_Toc160533074)

**[Table S4](#_Toc160533075)** [The results of model simulation 9](#_Toc160533075)

**[Table S5](#_Toc160533076)** [Comparing descriptives of observed and imputed data 10](#_Toc160533076)

[SUPPLEMENTARY FIGURES 12](#_Toc160533077)

**[Fig. S1](#_Toc160533078)** [Complete case: Variable importance provided by the random forest model. 12](#_Toc160533078)

[SUPPLEMENTARY REFERENCES 13](#_Toc160533079)

## SUPPLEMENTARY METHODS

### Outliers, missingness, and imputation

We inspected outliers and missingness of potential variables of use. Single-tone outliers that lie beyond 3 standard deviations (SD) from the variable means were identified and set to missing. All raw variables have a missingness lower than 60%. The Little’s test was conducted and confirmed that the missingness pattern of the data ($\chi^{2}$= 22,947.10, $P$=1.000) was tested highly likely to be missing completely at random[1]. Therefore, the Expectation-Maximization (EM) technique was used to treat missingness. By the assumption of multivariate normal distribution of the data and the criterion of maximum log-likelihood, taking $n$ as the number of iterations, EM iteratively computes a conditional probability distribution $q^{n+1}$ over completions of missing data given the model parameter $\theta^{n}$ obtained from the observed data structure (E-step) and re-estimates $\theta^{n+1}$ from the preliminary complete data via $q^{n+1}$ and $\theta^{n}$ (M-step) until model convergence achieved. The potentially introduced bias was inspected by non-parametric paired test of median difference (i.e., Wilcoxon signed rank tests for continuous and ordinal variables, McNemar’s tests for binary variables, and McNemar-Bowker symmetry tests for multinominal variables)[2]. No median difference was found, indicating the validity of the complete dataset for further analyses. The percentage of missingness, summary statistics before and after imputation were list in Table S5.

### Sample size and power of the study

In the Model_MLR_, to calculate a priori sample, the sjstats package was used[3]. A conservative interclass correlation coefficient (ICC) was assumed 0.05; the maximum number of clusters in the most extended multivariate model was approximated 45. The model design embraced a significance level of 0.05, secured the power at 0.80, and aimed to detect a small-to-medium effect size (Cohen’s $d$=0.30)[4]. To be statistically empowered, 546 observations was the minimal requirement that can be fully fulfilled with numerous observations in GROUP. As for the Model_RF_, a general rule of thumb is that the number of predictors counts no higher than 10% of the observations, and the 10% ratio is preferably lower[5]. Thus, 89 predictors (=1,119$\times$80%$\times$10%) at maximum can be included in the model.

### Prediction modelling

Given the multinomial outcome (i.e., mSI cluster), two methods were chosen accordingly for prediction. In the traditional approach, potential predictors were selected by reaching a predictor $P$-value$\leq$0.2 in the univariate multinomial mixed-effect model with a random effect of four medical centres (only indexed variable included as predictor). We raised the $P$-value threshold of inclusion, as we did not want to lose any potential important factors under the influence of other variables included in the model. The multivariate model used multinomial logistic regression (MLR) with medical centres as one of the fixed covariates. The predictors were evaluated and chosen by backward selection and variable exclusion was assessed jointly by overall model performance statistics including Akaike Information Criterion (AIC), log-likelihood, and the analysis of variance (ANOVA test) and clinical knowledge. Of note, due to the lack of mature and sophisticated automated techniques for selecting variables in multi-class outcome models, and the fact that this area is still under active research, we opted to manually select variables. The absence of multicollinearity was investigated (correlation coefficient$\geq$0.8) prior to the model finalization. Bootstrapping with 1000 replications was used for internal validation.

In the machine learning approach, random forest (RF) was chosen, among all algorithm candidates such as decision tree, K-Nearest Neighbour and Support Vector Machine, as RF is frequently used and adopts ensemble learning (i.e., developing a large number of diversified uncorrelated decision trees). It gives prediction based on the majority votes of the underlying trees and is less prone to overfitting. To construct Model_RF_, the dataset was randomly split into training and testing sets by an 80/20% ratio. Synthetic minority oversampling technique (SMOTE) was applied to balance the outcome classes, using the DMwR package[6,7]. Then, a RF algorithm enhanced by inner repeated cross-validation (i.e., the process of training data split into three subsets for training and validating repeats ten times) was developed. Feature selection in the initial runs was guided by feature importance indices. Afterwards, variables were visualized in a descending order. To avoid excluding informative variables, backward selection was used. Subsequently, hyperparameter was tuned via grid search. The final Model_RF_ was next validated on the testing set.

Complete cases analyses were conducted. Only participants with complete data for the variables included in the models were used for assessing the model accuracy. Whenever feasible, models were re-estimated to ensure their validity when built on imputed data.

####

## SUPPLEMENTARY RESULTS

### Complete-case analyses

There were 251 and 120 participants with complete data for Model_MLR_ and Model_RF_ respectively. The model accuracies with complete cases were 45.83% (95%CI:[36.71%,55.17%]; $P$=0.993) for Model_MLR_ and 91.24% (95%CI:[87.03%,94.43%]; $P$<0.001) for Model_RF_. The Model_MLR_ could not be re-estimated properly as the number of complete cases was too small to provide sensible estimates. On the contrary, the re-estimated Model_RF_ consistently showed important factors such as WHOQOL-BREF domain scores and symptom severity (positive, core negative and depression distress level) (Supplementary Fig. S1).

## SUPPLEMENTARY CLINICAL ILLUSTRATION

### A real case example

Model implementation in clinical practices is demonstrated. Hypothetically, Ms. Z came to the clinic and got examinations done. The collected data were entered into the models. The Model_RF_ predicts Ms. Z will be in the MH group in 3 years as well with a probability of 0.79 compared to the probability in LL (0.03), HL (0.12) and HH (0.06). At the three-year follow-up, Ms. Z is partitioned to MH as predicted.

| Name: Ms. Z | First visit (T_0_) Baseline | Any time point (T_x_)  between baseline and three-year |
| --- | --- | --- |
|  |  |  |
| Age | 35 |  |
| Age of onset | 31 |  |
| Duration of psychosis | 4 years |  |
| IQ | 89 |  |
| CTQ-total | 1.68 |  |
| PAS-overall | 2.68 |  |
| PRS_SCZ_ | -8.22 |  |
| PANSS-Positive symptoms | 7 |  |
| PANSS-Core negative symptoms | 5 |  |
| CAPE-depressive symptoms-frequency | 0.38 |  |
| CAPE-depressive symptoms-distress level | 2.00 |  |
| GAF-disability | 90 |  |
| GAF-symptom | 80 |  |
| Remission | No | Yes, longer than 6 months |
| CAN-met needs | 1 |  |
| WHOQOL-BREF-environment domain | 3.57 |  |
| WHOQOL-BREF-social domain | 4.00 |  |
| WHOQOL-BREF-physical domain | 3.57 |  |
| WHOQOL-BREF-psychology domain | 3.33 |  |
| Change of alcohol units consumed per week | 0 | 1 |
| Change of cigarette units consumed per week | 0 | 0 |
| Change of total week with cannabis use in the past 12 months | 0 | 0 |
| Predicted mSI group in 3-year by Model_RF_ | MH | MH |

*Note*: The data of Mr. Z listed in the table is the data from a participant in the GROUP database. The variables measuring changes between the baseline and Year-3 were set to ‘no change’ at the baseline and might be updated at any time between the baseline and three-year if any change has occurred. *Abbreviations*: CTQ, Childhood Trauma Questionnaire, Dutch Version; PAS, Premorbid Adjustment Score; PANSS, Positive And Negative Syndrome Scale; GAF, Global Assessment of Functioning; CAN, Camberwell Assessment of Need; WHOQOL-BREF, the abbreviated version of the preWorld Health Organization Quality of Life; mSI, multidimensional social inclusion.

## SUPPLEMENTARY TABLES

**Table S1** Thirteen subscales used for constructing outcome multidimensional social inclusion

| Questionnaire | Subscales | | Explanation |
| --- | --- | --- | --- |
| SFS | 1 | Independence performance | The performance of using a set of skills for independent living, such as if a person often or rarely takes public transportation independently. |
|  | 2 | Independence competence | The capability of using a set of skills for independent living, such as if a person is able or not to take public transportation independently. |
|  | 3 | Occupation | The engagement in the labour market or structured daily activities, such as if employed, job type, work hours, starting date of the permanent job/last employment no permanent job, rejected job application, if confident for a certain kind of job, frequency of job application. |
|  | 4 | Withdrawal | How socially engaged a person is, such as time of getting up every day, time spent alone, initiation of conversations, and social avoidance. |
|  | 5 | Interperson | Interpersonal behaviours such as number of friends/heterosexual contact, communication quality and easiness/difficulty of talking to people. |
|  | 6 | Recreation | The engagement of leisure activities such as hobbies and interests, including reading, gardening, knitting and so forth. |
|  | 7 | Prosociality | The engagement of a range of social activities, such as sport, exhibition and gatherings and so forth. |
| WHOQOL-BREF | 8 | QoL | Perceived quality of life |
|  | 9 | Health | Perceived overall health |
|  | 10 | Environment domain | Facets such as freedom and safety, home environment, financial resources, accessibility and quality of health and social care, opportunities for acquiring new information and skills, transport and so forth |
|  | 11 | Physical domain | Facets such as discomfort, rest, fatigue, mobility, work capacity, medication dependence and so forth |
|  | 12 | Psychological domain | Facets such as positive and negative feelings, self-esteem, bodily image, personal beliefs and so forth |
|  | 13 | Social domain | Social domain includes facets such as personal relationships, social support and sexual activity |

*Abbreviations*: SFS, social functioning scales; WHOQOL-BREF, the abbreviated version of World Health Organization Quality of Life; QoL, quality of life.

**Table S2** Statistical indexes of K-means clustering

| Cluster (K) | silhouette | duda | pseudot2 | Hartigan | gap indexes |
| --- | --- | --- | --- | --- | --- |
| 2 | 0.36 | 1.03 | -22.42 | 78.54 | 0.02 |
| 3 | 0.26 | 1.23 | -162.12 | 54.03 | -0.75 |
| 4 | 0.26 | 2.02 | -348.21 | 185.22 | -1.29 |
| 5 | 0.28 | 1.72 | -105.86 | 48.24 | -1.62 |
| 6 | 0.28 | 1.85 | -102.31 | 27.86 | -1.90 |
| 7 | 0.28 | 1.17 | -25.30 | 42.78 | -2.20 |
| 8 | 0.29 | 1.47 | -63.68 | 29.24 | -2.40 |
| 9 | 0.28 | 1.53 | -39.49 | 28.25 | -2.67 |
| 10 | 0.29 | 2.18 | -91.90 | 27.33 | -2.86 |
| Choice of K | 2 | 2 | 2 | 5 | 2 |

*Note*: In silhouette, duda, pseudot2 and gap indexes, the K of the highest index value should be chosen, while in the Hartigan index, the K of the lowest index value should be chosen.

**Table S3** Subgroup differences in multidimensional social inclusion by 13 subscales

| SFS |  |  |  |  |  |  | WHOQOL-BREF | |  |  |  |  |  |
| --- | --- | --- | --- | --- | --- | --- | --- | --- | --- | --- | --- | --- | --- |
| Subscale (7x) | mSI cluster | |  |  |  |  | Subscale (6x) | mSI cluster | |  |  |  |  |
| Independence performance |  | VLL | LL | HL | MH | HH | QoL |  | VLL | LL | HL | MH | HH |
|  | VLL |  | 1.000 | 0.000 | 0.000 | 0.000 |  | VLL |  | 0.000 | 0.000 | 0.000 | 0.000 |
|  | LL |  |  | 0.000 | 0.000 | 0.000 |  | LL |  |  | 1.000 | 0.000 | 0.000 |
|  | HL |  |  |  | 0.000 | 0.002 |  | HL |  |  |  | 0.000 | 0.000 |
|  | MH |  |  |  |  | 0.000 |  | MH |  |  |  |  | 0.000 |
|  | HH |  |  |  |  |  |  | HH |  |  |  |  |  |
| Independence competence |  | VLL | LL | HL | MH | HH | Health |  | VLL | LL | HL | MH | HH |
|  | VLL |  | 1.000 | 0.000 | 0.000 | 0.000 |  | VLL |  | 0.000 | 0.002 | 0.000 | 0.000 |
|  | LL |  |  | 0.000 | 0.000 | 0.000 |  | LL |  |  | 0.000 | 0.000 | 0.000 |
|  | HL |  |  |  | 1.000 | 0.000 |  | HL |  |  |  | 0.000 | 0.000 |
|  | MH |  |  |  |  | 0.000 |  | MH |  |  |  |  | 0.000 |
|  | HH |  |  |  |  |  |  | HH |  |  |  |  |  |
| Occupation |  | VLL | LL | HL | MH | HH | Environment domain |  | VLL | LL | HL | MH | HH |
|  | VLL |  | 0.000 | 0.000 | 0.000 | 0.000 |  | VLL |  | 0.003 | 0.065 | 0.000 | 0.000 |
|  | LL |  |  | 1.000 | 1.000 | 0.000 |  | LL |  |  | 1.000 | 0.000 | 0.000 |
|  | HL |  |  |  | 0.211 | 0.000 |  | HL |  |  |  | 0.000 | 0.000 |
|  | MH |  |  |  |  | 0.000 |  | MH |  |  |  |  | 0.000 |
|  | HH |  |  |  |  |  |  | HH |  |  |  |  |  |
| Withdrawal |  | VLL | LL | HL | MH | HH | Physical domain |  | VLL | LL | HL | MH | HH |
|  | VLL |  | 0.161 | 0.000 | 0.000 | 0.000 |  | VLL |  | 0.000 | 0.004 | 0.000 | 0.000 |
|  | LL |  |  | 0.000 | 0.000 | 0.000 |  | LL |  |  | 0.818 | 0.000 | 0.000 |
|  | HL |  |  |  | 0.970 | 0.000 |  | HL |  |  |  | 0.000 | 0.000 |
|  | MH |  |  |  |  | 0.000 |  | MH |  |  |  |  | 0.000 |
|  | HH |  |  |  |  |  |  | HH |  |  |  |  |  |
| Interperson |  | VLL | LL | HL | MH | HH | Psychological domain |  | VLL | LL | HL | MH | HH |
|  | VLL |  | 1.000 | 0.000 | 0.000 | 0.000 |  | VLL |  | 0.000 | 0.000 | 0.000 | 0.000 |
|  | LL |  |  | 0.000 | 0.000 | 0.000 |  | LL |  |  | 1.000 | 0.000 | 0.000 |
|  | HL |  |  |  | 0.000 | 0.000 |  | HL |  |  |  | 0.000 | 0.000 |
|  | MH |  |  |  |  | 0.000 |  | MH |  |  |  |  | 0.000 |
|  | HH |  |  |  |  |  |  | HH |  |  |  |  |  |
| Recreation |  | VLL | LL | HL | MH | HH | Social domain |  | VLL | LL | HL | MH | HH |
|  | VLL |  | 0.050 | 0.000 | 0.000 | 0.000 |  | VLL |  | 0.000 | 0.000 | 0.000 | 0.000 |
|  | LL |  |  | 0.000 | 0.000 | 0.000 |  | LL |  |  | 1.000 | 0.000 | 0.000 |
|  | HL |  |  |  | 0.000 | 1.000 |  | HL |  |  |  | 0.000 | 0.000 |
|  | MH |  |  |  |  | 0.000 |  | MH |  |  |  |  | 0.000 |
|  | HH |  |  |  |  |  |  | HH |  |  |  |  |  |
| Prosociality |  | VLL | LL | HL | MH | HH |  |  |  |  |  |  |  |
|  | VLL |  | 0.032 | 0.000 | 0.000 | 0.000 |  |  |  |  |  |  |  |
|  | LL |  |  | 0.000 | 0.000 | 0.000 |  |  |  |  |  |  |  |
|  | HL |  |  |  | 0.000 | 0.034 |  |  |  |  |  |  |  |
|  | MH |  |  |  |  | 0.000 |  |  |  |  |  |  |  |
|  | HH |  |  |  |  |  |  |  |  |  |  |  |  |

*Note*: The table reports the adjusted $\text{P}$-values between two mSI subgroups from Dunn’s multiple comparison.

*Abbreviations*: VLL, “very low/very low” mSI subgroup characterized by the lowest levels of social functioning and quality of life while the quality of life is even worse; LL, “low/low” mSI subgroup featured by low levels of social functioning and quality of life but moderately better quality of life; HL, “high/low” mSI subgroup with a high social functioning but low quality of life; MH, “medium/high” mSI subgroup with a medium level social functioning but a relatively high level of quality of life; HH, “high/high” mSI subgroup featured by the highest level of both social functioning and quality of life.

**Table S4** The results of model simulation

| Simulation | HL% | Model_MLR_ accuracy | Model_RF_ accuracy |
| --- | --- | --- | --- |
| % of the observed patient population | Mean $\pm$ SD | Mean $\pm$ SD | Mean $\pm$ SD |
| 30 | 49.27 $\pm$ 2.50 | 59.12% $\pm$ 2.50% | 92.29% $\pm$ 1.34% |
| 50 | 49.29 $\pm$ 1.84 | 59.16% $\pm$ 1.76% | 92.27% $\pm$ 0.98% |
| 70 | 49.25 $\pm$ 1.48 | 59.12% $\pm$ 1.43% | 92.27% $\pm$ 0.82% |
| 80 | 49.24 $\pm$ 1.34 | 59.13% $\pm$ 1.29% | 92.26% $\pm$ 0.74% |
| 90 | 49.24 $\pm$ 1.21 | 59.13% $\pm$ 1.20% | 92.26% $\pm$ 0.67% |

*Abbreviations*: HL, one of the multidimensional social inclusion subgroups demonstrating high social functioning and low quality of life; MLR, multinomial logistic regression; RF, random forest; SD, standard deviation.

**Table S5** Comparing descriptives of observed and imputed data

| N=1,119 | Observed data |  | Complete data |
| --- | --- | --- | --- |
|  | Missingness% | Median [IQR] / N (%) | Median [IQR] / N (%) |
| *A. Model predictor ^d^* |  |  |  |
| Medical centre | 0.00 |  |  |
| Amsterdam |  | 283 (25.29) | 283 (25.29) |
| Groningen |  | 287 (25.65) | 287 (25.65) |
| Maastricht |  | 306 (27.35) | 306 (27.35) |
| Utrecht |  | 243 (21.72) | 243 (21.72) |
| Age (year) | 0.27 | 26.00 [22.00, 32.00] | 26.00 [22.00, 32.00] |
| Gender: female | 0.00 | 267 (23.86) | 267 (23.86) |
| Ethnicity: non-Caucasian | 3.40 | 222 (20.54) | 256 (22.88) |
| PRS_SCZ_ | 36.64 | -3.45 [-7.11, 0.97] | -3.16 [-5.92, 0.00] |
| PAS-overall | 15.91 | 1.89 [1.37, 2.53] | 1.95 [1.42, 2.47] |
| CTQ-total | 32.53 | 1.48 [1.24, 1.84] | 1.53 [1.32, 1.80] |
| IQ | 6.88 | 94.00 [83.00, 105.00] | 94.00 [83.50, 105.00] |
| Age of onset (year) | 0.18 | 21.00 [18.00, 26.00] | 21.00 [18.00, 26.00] |
| Duration of psychosis (year) | 4.74 | 3.00 [1.00, 6.00] | 3.00 [1.00, 6.00] |
| Diagnosis: affective |  | 121 (10.81) | 121 (10.81) |
| Positive symptoms | 8.76 | 12.00 [9.00, 18.00] | 13.00 [9.00, 17.50] |
| Core negative symptoms | 8.13 | 9.00 [6.00, 14.00] | 10.00 [6.00, 13.00] |
| Depressive symptom (frequency) | 21.18 | 0.88 [0.62, 1.38] | 1.00 [0.62, 1.29] |
| Depressive symptom (distress level) | 23.77 | 1.43 [1.00, 2.00] | 1.40 [1.00, 1.82] |
| GAF-disability | 12.15 | 55.00 [41.00, 65.00] | 54.00 [42.00, 65.00] |
| GAF-symptom | 11.80 | 55.00 [45.00, 65.00] | 55.00 [45.00, 65.00] |
| Remission (baseline to 3-year) ^a^ | 32.62 |  |  |
| No |  | 309 (40.98) | 365 (32.6) |
| Yes (less than 6 months) |  | 177 (23.47) | 464 (41.5) |
| Yes (over 6 months) |  | 268 (35.54) | 290 (25.9) |
| Current urbanicity | 33.24 |  |  |
| No to little |  | 309 (41.37) | 422 (37.7) |
| Moderate |  | 73 (9.77) | 238 (21.3) |
| Strong to very strong |  | 365 (48.86) | 459 (41.0) |
| Work: Full time or part time | 9.92 | 514 (51.00) | 615 (55.0) |
| Work payment | 9.92 |  |  |
| None |  | 414 (41.10) | 429 (38.34) |
| Paid |  | 329 (32.63) | 404 (36.10) |
| Voluntary |  | 112 (11.11) | 132 (11.80) |
| Mixed |  | 105 (10.42) | 104 (9.29) |
| Unknown |  | 50 (4.96) | 50 (4.47) |
| Number of met needs | 11.08 | 4.00 [2.00, 5.00] | 4.00 [2.00, 5.00] |
| WHOQOL-BREF (baseline) |  |  |  |
| Environment domain | 11.62 | 3.57 [3.14, 4.00] | 3.57 [3.14, 3.99] |
| Physical domain | 11.62 | 3.43 [3.00, 3.86] | 3.43 [3.00, 3.86] |
| Psychosocial domain | 11.62 | 3.33 [2.83, 3.83] | 3.33 [2.83, 3.83] |
| Social domain | 11.62 | 3.33 [2.67, 4.00] | 3.33 [2.67, 3.67] |
| Duration of using antipsychotics (year) | 17.78 | 1.00 [0.00, 10.00] | 2.00 [0.00, 12.00] |
| Chlorpromazine equivalents antipsychotic dose ^b,c^ | 58.36 |  |  |
| Drastic reduction |  | 88 (13.48) | 252 (22.52) |
| Moderate reduction |  | 100 (15.31) | 308 (27.52) |
| No change |  | 114 (17.46) | 114 (10.19) |
| Moderate increase |  | 76 (11.64) | 202 (18.05) |
| Drastic increase |  | 88 (13.48) | 243 (21.72) |
| Awareness of antipsychotic use: (Become) aware ^b,d^ | 36.19 | 601 (84.17) | 1,003 (89.64) |
| Change of alcohol units consumed per week ^b^ | 69.35 | 0.00 [-2.00,2.00] | 0.00 [-2.00, 3.00] |
| Change of cigarette units consumed ^b^ | 69.26 | 0.00 [-1.00, 3.00] | 0.00 [-1.00, 4.00] |
| Change of total week with cannabis consumption in past 12 months ^b^ | 68.81 | 0.00 [0.00, 0.00] | 0.00 [0.00, 2.00] |
| *B. Outcome (subscales to construct mSI)* |  |  |  |
| SFS (3-year) |  |  |  |
| Independence performance | 33.69 | 109.00 [105.50, 118.50] | 109.80 [107.00, 114.50] |
| Independence competence | 33.69 | 114.00 [107.00, 123.00] | 113.30 [110.00, 117.50] |
| Occupation | 33.60 | 112.50 [97.50, 116.00] | 107.70 [106.70, 112.50] |
| Withdrawal | 33.51 | 104.50 [96.50, 110.00] | 104.40 [100.00, 110.00] |
| Interperson | 33.51 | 124.00 [105.00, 145.00] | 124.00 [111.00, 145.00] |
| Recreation | 33.60 | 116.50 [103.00, 126.50] | 115.00 [109.50, 121.50] |
| Prosociality | 33.51 | 114.50 [101.50, 125.00] | 113.10 [108.50, 119.00] |
| WHOQOL-BRE (3-year) |  |  |  |
| QoL | 32.89 | 4.00 [3.00, 4.00] | 4.00 [4.00, 4.00] |
| Health | 32.89 | 4.00 [3.00, 4.00] | 4.00 [3.00, 4.00] |
| Environment domain | 33.15 | 3.71 [3.29, 4.14] | 3.73 [3.57, 3.93] |
| Physical domain | 33.15 | 3.71 [3.29, 4.14] | 3.64 [3.43, 3.86] |
| Psychosocial domain | 33.07 | 3.50 [3.17, 4.00] | 3.51 [3.33, 3.83] |
| Social domain | 33.24 | 3.33 [2.67, 4.00] | 3.36 [3.00, 3.67] |

Note:

^a^ Remission is a longitudinal variable that recorded a patient’s remission status between the baseline and 3-year follow-up.

^b^ The variable indicates change from baseline to three-year follow-up. For the continuous variables, a positive, negative and 0 value implies an increased, decreased and no change in use.

^c^ Specifically, the antipsychotic dose excluding no dose change (0 value) were divided by 25 percentiles into four groups which were denoted as drastic reduction, moderate reduction, moderate increase, and drastic increase.

^d^ Being (or becoming) aware of antipsychotic use refers to the patients who are aware of them using antipsychotic at baseline and 3-year follow-up (or being unaware at the baseline and be).

^e^ For explanation: a higher value of PRS_SCZ_ (polygenic risk score for schizophrenia) indicates a stronger genetic predisposition of developing SSD (schizophrenia spectrum disorder); PAS (Premorbid Adjustment Score) has a 7-point scale for each item in different age periods (0 healthiest adjustment, 6 lowest adjustment) and PAS-overall is an average score retrospectively measured for childhood (before age 12), early adolescence (12-16) and late adolescence1(6-19) prior to the disease onset where a higher PAS-overall reflects a worse premorbid adjustment; CTQ (Childhood Trauma Questionnaire, Dutch Version) has a 5-point scale for each item (1 never true, 5 very often true) in each subscale (i.e., emotional abuse, physical abuse, sexual abuse, emotional neglect and physical neglect) and CTQ-total is an average score of all subscales where a higher CTQ-total reflect a severer childhood adversity; a higher IQ indicates a better cognitive ability; a higher value of positive symptoms or core negative symptoms (i.e., Blunted affect, Emotional withdrawal, Poor rapport, Lack of spontaneity, Active social avoidance) from PANSS (Positive And Negative Syndrome Scale) indicates severer symptoms; depressive symptoms (frequency and distress level) from CAPE (community assessment of psychic experience) have a 4-point scale (1 never, 4 nearly always; 1 not distressed, 4 very distressed); a higher value of GAF (Global Assessment of Functioning)-disability or -symptoms indicates less severe disability or symptoms (i.e., less impairment); from Camberwell Assessment of Need (CAN), a higher number of met needs suggests a better-fulfilled individual needs; four domains of WHOQOL-BREF (the abbreviated version of the World Health Organization Quality of Life) has a 5-point scale for each domain item, and each domain score ranges from 1 (very dissatisfied) to 5 (very satisfied); a higher value of a subscale of *SFS (Social Functioning Scale)*, a higher level of social functioning level of the specific subscale area.

*Abbreviations*: IQR, interquartile range; mSI, multidimensional social inclusion.

## SUPPLEMENTARY FIGURES


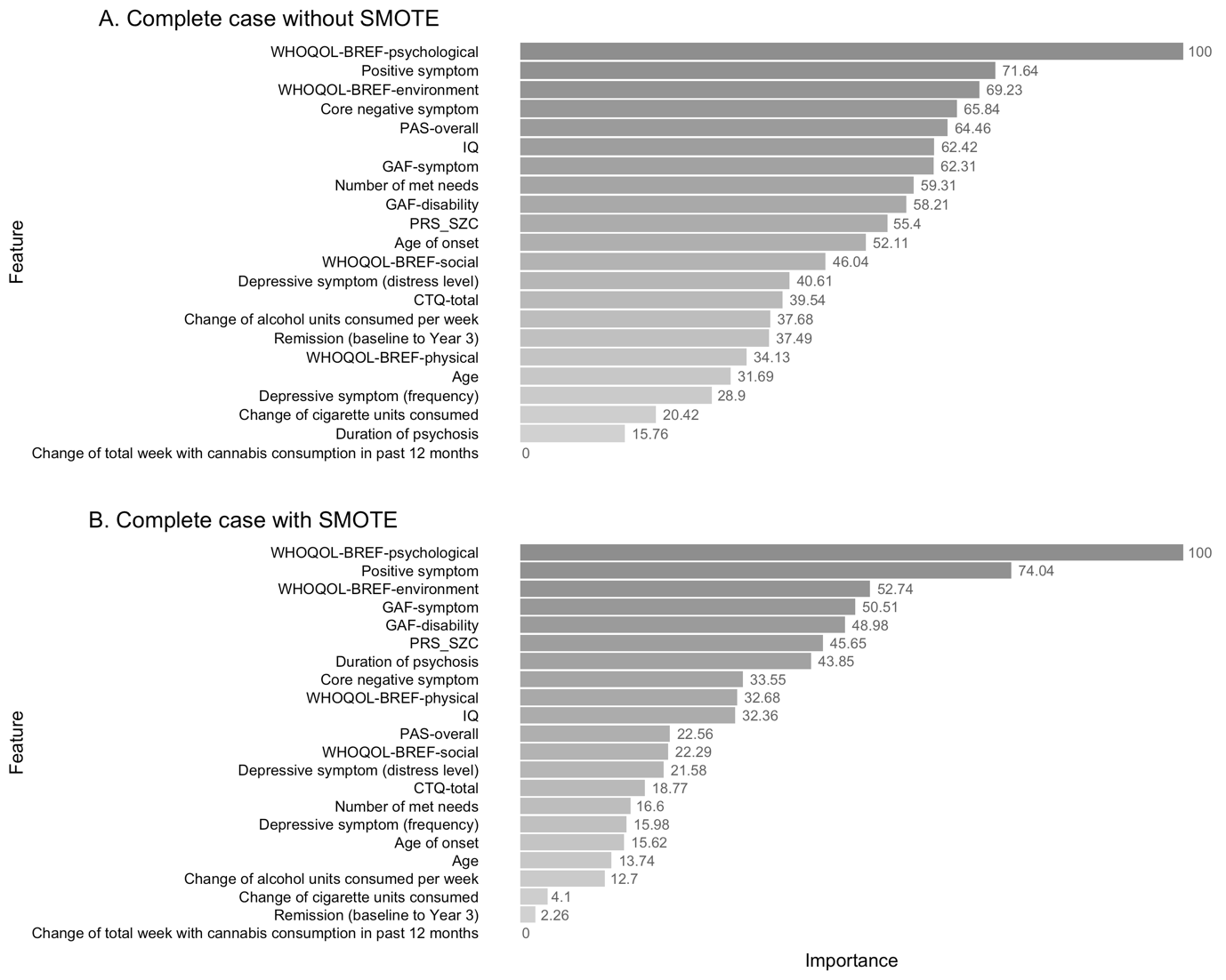


b. Complete case with SMOTE

a. Complete case without SMOTE

**Fig. S1** Complete case: Variable importance provided by the random forest model.

*Abbreviations*: WHOQOL-BREF, the abbreviated version of World Health Organization Quality of Life; CTQ, Childhood Trauma Questionnaire, Dutch Version; GAF, global assessment of functioning; PAS, premorbid adjustment score; PRS_SCZ_, polygenic risk score for schizophrenia.

## SUPPLEMENTARY REFERENCES

1. Little RJA (1988) A Test of Missing Completely at Random for Multivariate Data with Missing Values. Journal of the American Statistical Association 83 (404):1198-1202. doi:10.1080/01621459.1988.10478722

2. Dong Y, Peng C-YJ (2013) Principled missing data methods for researchers. SpringerPlus 2 (1):222. doi:10.1186/2193-1801-2-222

3. Lüdecke D (2021) sjstats - Collection of Convenient Functions for Common Statistical Computations. <https://www.rdocumentation.org/packages/sjstats/versions/0.18.1>. Accessed 26 May 2021

4. Cohen J (1988) Statistical Power Analysis for the Behavioural Sciences. Laurence Erlbaum Associates, Hillsdale, NJ

5. Domingos P (2012) A few useful things to know about machine learning. Communications of the ACM 55 (10):78-87

6. Fernandez A, Garcia S, Herrera F, Chawla NV (2018) SMOTE for Learning from Imbalanced Data: Progress and Challenges, Marking the 15-year Anniversary. Journal of Artificial Intelligence Research 61:863-905. doi:10.1613/jair.1.11192

7. Torgo L (2010) Data Mining with R, learning with case studies Chapman and Hall/CRC
